# Incidental Finding of a Superdominant Left Anterior Descending Artery in an 80-Year-Old Woman: Case Report and Literature Review

**DOI:** 10.7759/cureus.96951

**Published:** 2025-11-16

**Authors:** Ahmed Maalej, Usama Selim

**Affiliations:** 1 College of Medicine, University of Sharjah, Sharjah, ARE; 2 Radiology, University Hospital Sharjah, Sharjah, ARE

**Keywords:** anatomy, coronary artery, coronary ct angiogram, incidental finding, posterior descending artery, radiology, type iv left anterior descending artery

## Abstract

We report a case of an 80-year-old woman who presented to the hospital with sudden-onset palpitation, where, during an out-patient clinic follow-up, we discovered a rare coronary artery finding on radiological imaging, and to the best of my research, the first reported case in the United Arab Emirates. We discovered during a CT angiography (CTA) scan a Type IV left anterior descending (LAD) artery, where the artery continues around the apex of the heart and ascends within the posterior interventricular groove as the posterior dominant artery. Since the encounter at the emergency room, our patient, who is dependent on her family members for most activities of daily living, has not experienced another onset of palpitation. She regularly visits her doctor, is compliant with her medications, and maintains a healthy lifestyle, following up with her cardiologist on a monthly basis.

## Introduction

A superdominant left anterior descending (LAD) artery is a rare radiological finding; only 19 case reports have been discussed and submitted within the literature as of 2018 [1]. Up to 2% of individuals have some sort of variations in the anatomy and layout of coronary arteries with notable differences in origin, course, or supply of either a single coronary or multiple coronary arteries [2]. These variations can be classified into benign and malignant, where the benign variation is not associated with increased likelihood or severity of a any particular cardiac event. These variants discovered incidentally during CT angiography (CTA) scan or interventional angiography [1,2]. The significance of this finding is crucial as our patient, with this type of artery and her risk factors for cardiac event such as elderly age, obesity, immobility, and diabetes, could suffer from a more severe form of a cardiac event.

## Case presentation

An 80-year-old woman presented to the emergency department with sudden-onset palpitation and anxiety that started an hour prior while the patient was at rest with no precipitating factors. This was the first time the patient experienced such palpitations, and she denied the typical associated symptoms such as syncope, chest pain, shortness of breath, nausea, vomiting, dizziness, and sweating. The palpitation was constant and did not change in severity.

**Figure 1:**
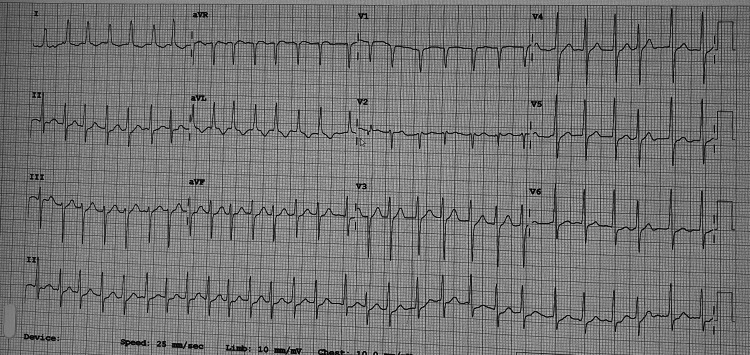
ECG taken as soon as the patient arrived at the hospital Heart rate was calculated by the machine to be 172 beats per minute with a PR interval of 87 milliseconds (normal: 120-200) and a QT interval of 266 milliseconds (normal: 360-460). The cardiologist concluded this ECG study with supraventricular tachycardia with left ventricular hypertrophy with secondary repolarization abnormality. ECG: Electrocardiogram

Vitals measured during triage showed a temperature of 36.7°C, heart rate of 146 beats per minute, blood pressure of 146/86 mmHg, respiratory rate of 20 breaths per minute, and oxygen saturation of 97% on room air. A bedside electrocardiogram (ECG) revealed paroxysmal atrial fibrillation at a rate of 172, as seen below in Figure 1. Medical history was significant for a 15-year history of hypertension, prediabetes, and hyperlipidemia. Medical history was also significant for osteoarthritis, right eye cataract, vitamin D deficiency, and spinal stenosis. Surgical history includes appendectomy, cholecystectomy, and left oophorectomy. According to her family member who presented with her, she regularly followed up with her doctors and was compliant with her medications.

Sinus rhythm was restored in the emergency room using carotid pressure and amiodarone infusion. Standard blood investigations were done, which showed decreased clotting time, low sodium, and elevated liver enzymes, as can be seen below in Table 1. The patient was started on glucose IV infusion to prevent dehydration and prescribed pantoprazole to prevent acid reflux, metoprolol tartrate to control her elevated blood pressure, and enoxaparin to prevent deep vein thrombosis (DVT) as she scored a score of 5 on the DVT prophylaxis risk assessment form.

**Table 1:** Blood investigatons done in the emergency room The text in bold signify abnormally high or low values. RBC: Red blood cell count; HCT: Hematocrit; MCV: Mean cell volume; WBC: White blood cell count; CRP: C-reactive protein; PT: Prothrombin time; INR: International normalized ratio; BUN: Blood urea nitrogen; ALT: Alanine aminotransferase; AST: Aspartate aminotransferase; ALP: Alkaline phosphatase; GGT: Gamma glutamyl transferase

Investigation	Result	Unit	Reference Range	Interpretation
RBC	4.63	x10^12^/L	3.8–4.8	
Hemoglobin	13.6	g/dL	12–15	
HCT	41.3	%	36–46	
MCV	89.3	fL	83–101	
Platelet count	208	x10^9^/L	150–410	
WBC	8.05	x10^9^/L	4–10	
Neutrophil	79.9	%	40–80	
Lymphocyte	13.9	%	20–40	
Monocyte	5.6	%	2–10	
CRP	<4.00	mg/L	0–10	
PT	11.8	seconds	12.5–16.1	Low
INR	0.85		0.87–1.33	Low
Sodium	135	mmol/L	136–145	Low
Potassium	3.51	mmol/L	3.5–5.1	
Magnesium	0.8	mmol/L	0.66–1.07	
Calcium	2.56	mmol/L	2.08–2.65	
Urea (BUN)	3.6	mmol/L	3.2–8.2	
Creatinine	71	umol/L	49–90	
Albumin	43	g/L	32–50	
ALT	65	U/L	14–59	High
AST	51	U/L	8–34	High
Bilirubin total	8.12	umol/L	5–21	
Bilirubin direct	2.47	umol/L	0–5	
Protein total	68	g/L	57–82	
ALP	104	U/L	46–116	
GGT	7	U/L	7–38	High

A comprehensive physical examination revealed an alert and conscious patient, non-toxic in appearance, not sweating, and oriented to time, place, and person, with a 15 out of 15 on Glasgow Coma Scale (GCS). She had no change in color such as pallor or jaundice cyanosis. The cardiovascular examination showed an irregular pulse with normal S1 and S2 sounds, no gallop, and no dilated neck veins. Her chest was clear with no added sounds. Her abdomen was lax, soft, and tender. The patient had no lower limb edema, swelling, cellulitis, or signs of DVT. A comprehensive bedside nervous system examination was conducted where no signs of lateralization, ocular, cranial nerve, cerebellar, meningeal, or gait manifestations were present. The patient left the emergency room against medical advice; further blood investigations and monitoring could not be conducted.

**Figure 2:**
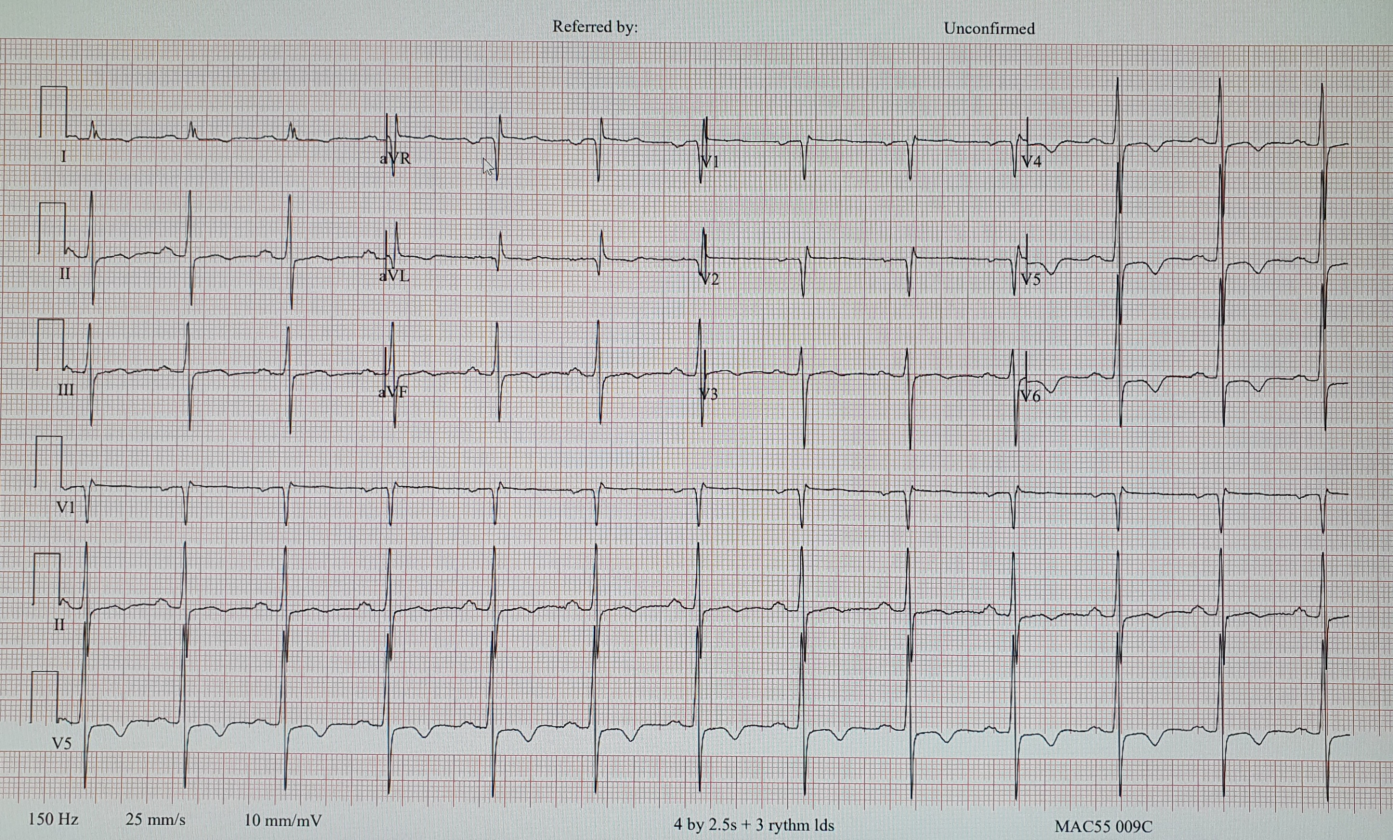
ECG done during a cardiology follow-up The patient's cardiologist concluded this ECG study with normal sinus rhythm with signs of ischemia in the lateral leads. ECG: Electrocardiogram

The patient visited a cardiologist two days later, where he recommended blood investigations, a new ECG, and a CTA scan. The results of the blood work showed significantly elevated troponin I and increased glycated hemoglobin and high-density lipoprotein (HDL) cholesterol levels, which can be found below in Table 2. Her new ECG, located below in Figure 2, showed improvement in her rhythm. The CTA scan concluded with mild calcified atherosclerosis stenosis of the LAD artery, calcification to the mitral and aortic valve, left atrial enlargement, and left ventricular hypertrophy.

**Table 2:** Investigations ordered by the patient's cardiologist The significance of these results were discussed during a cardiology follow-up two days after the onset of palpation. The text in bold signifies abnormally high or low values. CK-MB: Creatine kinase MB; LDL: Low-density lipoprotein; HDL: High-density lipoprotein

Investigation	Result	Unit	Reference Range	Interpretation
Creatine kinase	67	U/L	34–145	
CK-MB	1.67	Ug/L	0–5	
Troponin I high sensitivity	50.56	ng/L	0–47.3	High
Glycated hemoglobin	7.3	%	Normal: 4–5.6	Diabetic
Cholesterol Total	5.02	mmol/L	0–5.20	
Cholesterol LDL	2.94	mmol/L	0–4	
Cholesterol HDL	1.77	mmol/L	1.04–1.55	High
Cholesterol ratio	2.84		0–4	
Non-HDL cholesterol	3.25	mmol/L		
Triglycerides	0.65	mmol/L	0.4–1.8	
Thyroid-stimulating hormone	2.486	mIU/L	0.48–4.17	

**Figure 3:**
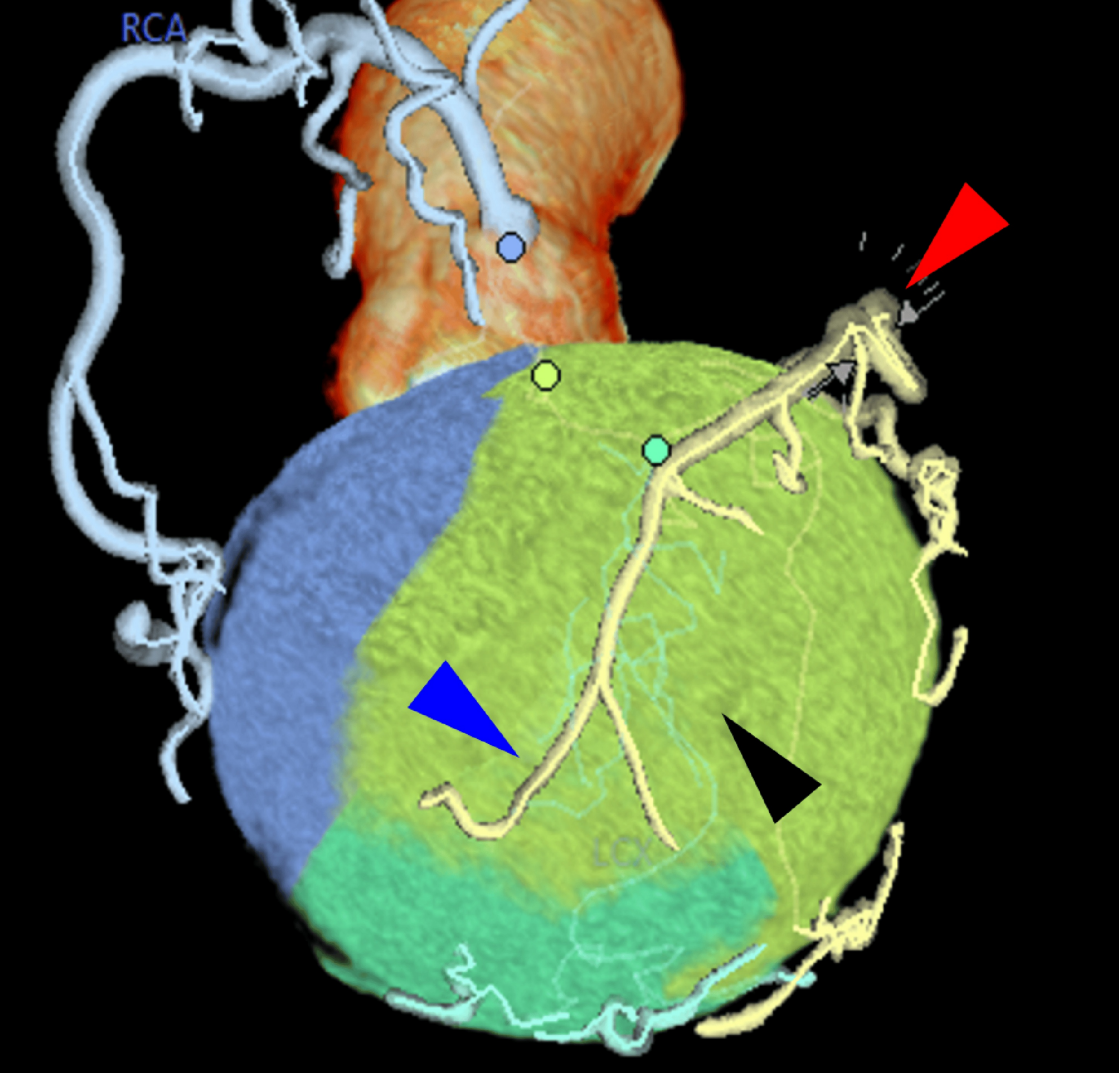
Left anterior oblique caudal to cranial view of the CTA scan with the perfusion Red arrowhead: LAD artery; Black arrowhead: Apex of the heart; Blue arrowhead: PDA ascending in the posterior interventricular groove CTA: CT angiography; LAD: Left anterior descending; PDA: Posterior descending artery

Also seen on the CTA scan is the topic of interest in this case report, a superdominant LAD artery, where it descends as usual in the anterior interventricular groove, wraps around the apex of the heart, and starts to ascend in the posterior interventricular groove as the posterior dominant artery, as seen in Figure 3. The patient's family understood the importance of this condition, and they assured us that compliance would be taken seriously and that she would be brought in regularly for her follow-ups with cardiology. She was prescribed nebivolol, amlodipine, and rivaroxaban by her cardiologist.

**Figure 4:**
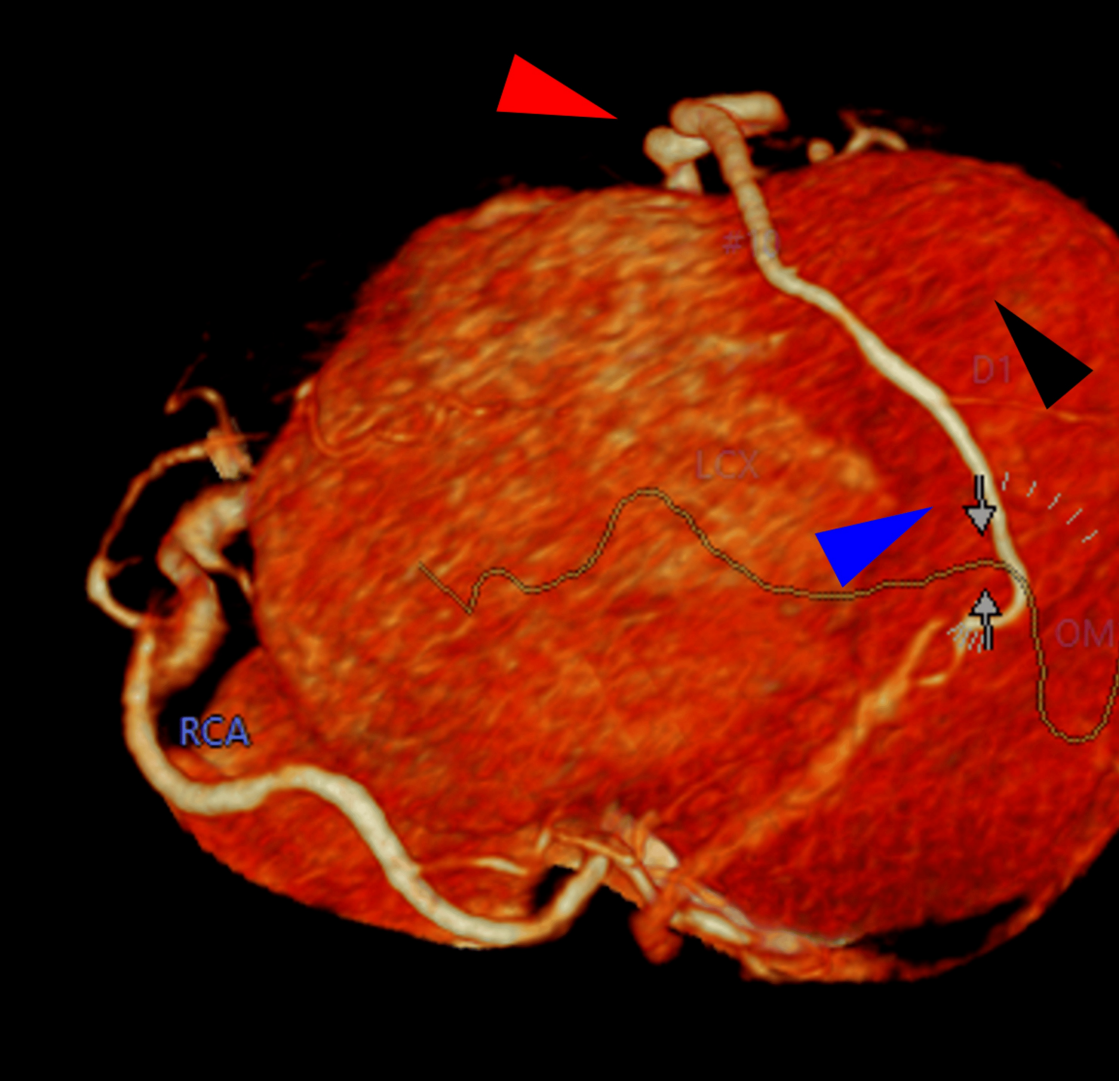
Right posterior oblique caudal to cranial view of the CTA scan Red arrowhead: LAD artery; Black arrowhead: Apex of the heart; Blue arrowhead: PDA ascending in the posterior interventricular groove CTA: CT angiography; LAD: Left anterior descending; PDA: Posterior descending artery

## Discussion

Introduction

The coronary artery anatomy is known to exhibit a large interindividual variability, similar to the characteristics of fingerprints, where it is difficult to find two people with the same pattern of coronary distribution. When the origin, course, or supply of a single coronary or multiple coronary arteries deviates from the standard, it is referred to as a coronary artery anomaly. 

In cardiology, if a coronary artery anomaly increases the likelihood of developing a myocardial infarction (MI), it is referred to as a malignant anomaly, as it is hemodynamically significant [3]. This anatomical variant is clinically significant as a coronary event of this variant of the LAD artery is linked with more severe ischemia and infarction with worse subsequent cardiogenic shock as the artery supplies a larger amount of anterior wall, septum, and inferior myocardium and is often more fatal compared to a normal variant LAD artery thrombosis [1,2].

Review of anatomy

Both the right and left coronary arteries emerge perpendicularly at the sinus portion of the aortic root. The left coronary artery, in the majority of cases, bifurcates into the anterior descending, which descends in the interventricular septum with further division into the diagonal arteries supplying the antero-lateral free wall of the left ventricle and the perforating arteries supplying two-thirds of the anterior ventricular septum and the left circumflex left atrioventricular (AV) groove [4]. In a minority of cases, approximately 7% of the collected specimens, the left coronary artery trifurcates, creating a blood vessel that descends between the anterior descending artery and left circumflex artery (LCX) [5]. The right coronary artery (RCA) runs in the right AV groove, giving rise to the origin of the descending coronary artery, also known as the posterior descending artery (PDA) [4]. 

One normal variant that Thiene et al. discuss in their article is when a patient has two LAD arteries, where one artery supplies the diagonal arteries and the other supplies the perforating arteries [4]. Thiene et al. also describe a normal variant without any MI implications, where the LCX originates directly from the aorta through its own separate orifice at the aortic sinus. A separate orifice of the left aortic sinus [4].

It is essential to note that the origin of the PDA is typically the RCA in approximately 85% of the population. In a minority of cases, it arises from the LCX [6]. A superdominant, also referred to as hyperdominant, LAD, refers to an anatomical variant where the PDA arises and takes origin at the apex of the heart from the LAD and continues to ascend within the posterior interventricular groove [1,6]. As of 2018, only 19 reported cases of superdominant LAD have been documented in the literature [1].

Epidemiology

It has been estimated that up to 2% of the general population has some coronary anomaly [1,2]. In the majority of cases, they are discovered incidentally during CTA scan or interventional angiography [1,2]. It is important to note that it is quite common to observe the continuation of the LAD artery around the apex, sometimes referred to as a Type III LAD [2]. Over the past years, it has been discovered that pathological deviation in coronary artery anatomy is the second most common cause of sudden cardiac death in athletes [3].

Clinical presentation

When a patient presents with a coronary event in a superdominant LAD, a cardiologist may notice minimal changes in the ECG, as the overall electrical changes that occur during anterior wall ischemia cancel those of the inferior wall ischemia and vice versa, as this artery supplies both the anterior and inferior myocardium. In some cases, a patient may present with combined ST elevation of anterior and inferior segments [1]. In other cases, a patient may present with an anterior wall MI with absent inferior wall electrical ST changes and vice versa [1]. Varga et al. described in their article that physicians should be more cautious when interpreting ECGs in these patients, as some of their ECGs may resemble those of pericarditis [3]. A superdominant LAD patient, in particular, would also present with increased severity of cardiovascular collapse and significantly poorer ventricular motion on cardiac echocardiogram [1]. 

Treatment

When this finding is discovered incidentally, cardiologists should spend more time educating their patients about the modifiable causes of coronary artery disease (CAD), such as increased body weight, sedentary lifestyle, smoking, a salty diet, high blood pressure, diabetes, and dyslipidemia, and emphasize the importance of patient compliance [7]. They should also keep an eye on and recommend increased physician follow-up for patients with non-modifiable factors of CAD, such as men, older patients, African American patients, and patients with strong family history [7]. Varga et al., in their article, recommend and suggest approaching this blood vessel anomaly as a double CAD and recommend coronary artery bypass surgery (CABG) instead of the typical intervention, stenting, used in patients with stable angina associated with CAD [3].

Embryology

The development of coronary artery vascularization begins when the epicardium spreads over the myocardium, a process that occurs in the same developmental step [8]. The vascularization process occurs in three steps: vasculogenesis, angiogenesis, and arteriogenesis [9]. The development of the coronary artery occurs in a distal to proximal fashion, ascending towards the aorta [8]. In experiments, disruption of myocardial cell polarity has been shown to influence the pattern and location of the coronary artery [8].

## Conclusions

This case report and rare finding remind us of the importance of physicians adjusting and increasing their flexibility when discussing the types of treatments that can be offered to patients and their prognosis, based on variations in anatomy and physiology. This case is also important as the disease of cardiovascular disease is the leading cause of death in the developed world. I hope this case reminds the reader, especially those born with a non-modifiable risk factor for any chronic condition, to increase their follow-ups with their family doctor and seek healthcare advice in the hopes of lowering any existing modifiable risk factors for cardiovascular disease.
